# Analyses of expressed sequence tags from the maize foliar pathogen *Cercospora zeae-maydis *identify novel genes expressed during vegetative, infectious, and reproductive growth

**DOI:** 10.1186/1471-2164-9-523

**Published:** 2008-11-04

**Authors:** Burton H Bluhm, Braham Dhillon, Erika A Lindquist, Gert HJ Kema, Stephen B Goodwin, Larry D Dunkle

**Affiliations:** 1 Department of Plant Pathology, University of Arkansas, Fayetteville, Arkansas, 72701, USA; 2 Department of Botany and Plant Pathology, Purdue University, West Lafayette, Indiana, 47907-2054, USA; 3 DOE-Joint Genome Institute, 2800 Mitchell Drive, Walnut Creek, CA 94598, USA; 4 Plant Research International B. V., P.O. Box 16, 6700 AA Wageningen, The Netherlands; 5 Crop Production & Pest Control Research Unit, USDA-Agricultural Research Service, Purdue University, West Lafayette, Indiana, 47907-2054, USA

## Abstract

**Background:**

The ascomycete fungus *Cercospora zeae-maydis *is an aggressive foliar pathogen of maize that causes substantial losses annually throughout the Western Hemisphere. Despite its impact on maize production, little is known about the regulation of pathogenesis in *C. zeae-maydis *at the molecular level. The objectives of this study were to generate a collection of expressed sequence tags (ESTs) from *C. zeae-maydis *and evaluate their expression during vegetative, infectious, and reproductive growth.

**Results:**

A total of 27,551 ESTs was obtained from five cDNA libraries constructed from vegetative and sporulating cultures of *C. zeae-maydis*. The ESTs, grouped into 4088 clusters and 531 singlets, represented 4619 putative unique genes. Of these, 36% encoded proteins similar (E value ≤ 10^-05^) to characterized or annotated proteins from the NCBI non-redundant database representing diverse molecular functions and biological processes based on Gene Ontology (GO) classification. We identified numerous, previously undescribed genes with potential roles in photoreception, pathogenesis, and the regulation of development as well as *Zephyr*, a novel, actively transcribed transposable element. Differential expression of selected genes was demonstrated by real-time PCR, supporting their proposed roles in vegetative, infectious, and reproductive growth.

**Conclusion:**

Novel genes that are potentially involved in regulating growth, development, and pathogenesis were identified in *C. zeae-maydis*, providing specific targets for characterization by molecular genetics and functional genomics. The EST data establish a foundation for future studies in evolutionary and comparative genomics among species of *Cercospora *and other groups of plant pathogenic fungi.

## Background

The fungal genus *Cercospora *represents a large and diverse group of plant pathogens that are distributed worldwide and infect numerous host species. Individual species of *Cercospora *are usually host specific, but collectively they infect remarkably diverse hosts. More than 3,000 species of *Cercospora *have been named [1], and they often are classified according to host association, e.g., *C. beticola *infects sugar beet (*Beta vulgaris*), *C. oryzae *infects rice (*Oryza sativa*), and *C. sorghi *infects sorghum (*Sorghum bicolor*). Most plant-pathogenic species of *Cercospora *enter host leaves through stomata, a process facilitated in part by the ability of elongating germ tubes to sense nearby stomata and reorient their direction of growth accordingly [2]. Upon reaching stomata, germ tubes differentiate into multilobed infection structures similar to appressoria, from which infectious hyphae penetrate mesophyll tissues. After a period of colonization, the fungus presumably adopts a necrotrophic growth habit, leading to the formation of expanding, necrotic lesions that coalesce in severe outbreaks, leading to a significant reduction in photosynthetic tissue, defoliation, and potentially premature death of the host plant. Reproduction and formation of secondary inocula occur in colonized tissue through the production of asexual spores (conidia) that infect neighboring plants after dispersal by wind and/or rain splash. Many diseases caused by *Cercospora *species occur periodically throughout the world as epidemics singly or as components of disease complexes [e.g., [3-5]], and for crops such as sugar beet, are major limitations for production [6]. Additionally, the possibility that *Cercospora *pathogens influence the distribution of plant species in natural ecosystems is a plausible but largely unexplored hypothesis.

*Cercospora zeae-maydis *is a foliar pathogen causing gray leaf spot of maize. Substantial economic losses from this disease occur annually throughout the Western Hemisphere. First discovered in 1924 in Illinois [7], *C. zeae-maydis *did not become an important pathogen of maize until the 1980s; by the mid-1990s, the fungus caused significant losses throughout the corn belt of the U.S. and it is now the most devastating foliar pathogen of maize in much of the world [8]. Colonization of leaves by the fungus causes distinctive rectangular lesions delineated by the major veins. When the incidence of infection is high before grain filling, the impaired photosynthetic capability of diseased leaves results in severe reductions in yield [8]. Management of *C. zeae-maydis *is especially difficult because commercial hybrids of maize lack effective resistance to gray leaf spot [8] and the fungus can survive between growing seasons in plant debris [9]. Exactly why *C. zeae-maydis *has ascended so rapidly as a pathogen of maize during the past two decades is not known, but speculation has linked the phenomenon to global climate change, the emergence of more virulent strains, and the increased practice of conservation tillage in maize production [8,10,11].

During pathogenesis, *C. zeae-maydis *and many other species of *Cercospora *produce the host non-specific phytotoxin cercosporin, a photosensitizing perylenequinone that causes lipid peroxidation and alters membrane permeability through the action of reactive oxygen species [12]. *Cercospora *pathogens protect themselves against the toxic effects of cercosporin through the functions of *CFP1*, which encodes an ABC transporter required for secretion [13], and *PDX1 *(formerly *SOR1*), a gene involved in the biosynthesis of pyridoxine (vitamin B6), which quenches singlet oxygen produced during the interaction of cercosporin with membranes [14]. Consistent with the production of many fungal secondary metabolites, cercosporin biosynthesis was recently demonstrated to result from the expression of genes organized in a cluster [15]. However, a molecular understanding is lacking to explain how *Cercospora *species integrate diverse environmental inputs to regulate cercosporin biosynthesis and the extent to which regulation is conserved throughout the genus. Typically, cultures of *C. zeae-maydis *producing asexual spores (conidia) do not produce cercosporin, suggesting that fungal development and secondary metabolism are antagonistic at some level. In culture, cercosporin biosynthesis is repressed by the presence of preferred nitrogen sources [16]; presumably, this regulation is a component of global changes in gene expression during nitrogen metabolite repression resulting from the actions of a homolog of the nitrogen-responsive transcription factor areA [17]. Additionally, the biosynthesis and activation of cercosporin require light [18], thus establishing an intriguing link between light and pathogenesis among *Cercospora *species.

The initial infection of maize leaves by *C. zeae-maydis *occurs in spring or early summer when propagules of the fungus that survived the winter in plant debris give rise to conidia that are dispersed onto leaves of young plants [8]. During colonization of leaf tissue, the fungus produces stromata that give rise to erumpent conidiophores bearing conidia that serve as secondary inocula [8]. Multiple cycles of secondary infection can occur when environmental conditions are favorable, leading to epidemic levels of infection. Somewhat surprisingly, *C. zeae-maydis *has not been demonstrated to reproduce sexually in laboratory conditions, and field populations appear to be largely clonal [19], although recent analyses of the distribution of mating type loci suggest the possibility of cryptic sex [20].

Despite the impact of *C. zeae-maydis *and other *Cercospora *species on global agriculture, very little information is available at the molecular level regarding how members of this genus regulate growth, development, and pathogenesis. The focus of this research was to generate a collection of expressed sequence tags (ESTs) from *C. zeae-maydis*, and to analyze their expression during defined stages of growth and development. To this end, we generated distinct cDNA libraries from vegetative cultures of *C. zeae-maydis *(vegetative libraries) as well as cultures producing conidia (sporulation libraries). Among 27,551 ESTs sequenced from both conditions, we identified 4619 unique sequences representing a broad range of molecular functions and biological processes. Of 4088 clusters containing two or more ESTs, 1436 were comprised of ESTs found exclusively in the sporulation libraries, whereas 1744 were unique to vegetative libraries. At least eight clusters encode putative photoreceptors and light-responsive genes, six are similar to genes regulating morphogenesis in other fungi, and 20 are implicated in host/pathogen interactions. The expression profiles of 15 clusters were characterized by real-time quantitative PCR, which largely confirmed their proposed roles in photoreception, conidiation, and pathogenesis. Furthermore, we identified *Zephyr*, a novel, highly transcribed member of the Ty3/Gypsy family of transposable elements. This research represents the first comprehensive EST sequencing project for *C. zeae-maydis*, and provides specific targets for subsequent studies in molecular genetics as well as a framework for future investigations into the evolution of pathogenesis among species of *Cercospora *and closely related genera.

## Methods

### Fungal strain and culture conditions

Wild-type *C. zeae-maydis *strain SCOH1-5, isolated from infected maize plants near South Charleston, Ohio in 1999, was used in all experiments. Cultures were maintained on V8 agar in constant darkness to provide conidia for inoculations. For library construction, the fungus was grown at 24°C on V8 agar, 0.2× potato dextrose agar (PDA; BD Biosciences, Sparks, MD), or 0.2× PDA supplemented with 10 mM ammonium phosphate. Cultures grown in constant light received 8–10 μE m^-2^s^-1 ^of illumination. To facilitate collection of fungal tissue from agar plates, conidial suspensions were inoculated onto cellophane membranes placed on the surface of the medium. Maize inbred line B73, which is highly susceptible to infection by *C. zeae-maydis*, was grown in a greenhouse and inoculated with conidia (10^5^/ml) with a fine-mist atomizer.

### RNA isolation, cDNA library construction and sequencing

RNA was extracted with Trizol reagent (Invitrogen; Carlsbad, CA) followed by purification with RNeasy Maxiprep columns (Qiagen; Valencia, CA). RNA quantity and quality were assessed with a Nano-Drop ND-1000 spectrophotometer (NanoDrop Technologies; Wilmington DE) and by gel electrophoresis following standard protocols [21]. Poly A+ RNA was isolated from total RNA for two *C. zeae-maydis *samples, sporulating or vegetative, using the Absolutely mRNA Purification kit (Stratagene; La Jolla, CA) and the manufacturer's instructions. cDNA synthesis and cloning were modified based on the "SuperScript plasmid system with Gateway technology for cDNA synthesis and cloning" (Invitrogen). Poly A+ RNA (1–2 μg), reverse transcriptase (SuperScript II; Invitrogen) and oligo dT-NotI primer (5'- GACTAGTTCTAGATCGCGAGCGGCCGCCCTTTTTTTTTTTTTTT -3') were used to synthesize first-strand cDNA. Second-strand synthesis was performed with *E. coli *DNA ligase, polymerase I, and RNaseH followed by end repair with T4 DNA polymerase. The *Sal*I adaptor (5'- TCGACCCACGCGTCCG and 5'- CGGACGCGTGGG) was ligated to the cDNA, digested with restriction enzyme *Not*I (New England Biolabs; Ipswitch MA), and subsequently size selected by gel electrophoresis (1.1% agarose). Two size ranges of cDNA were excised from the gel for each sample: 0.6 – 2 kb (vegetative library CBYB, sporulation library CBYG) and > 2 kb (vegetative library CBYC, sporulation library CBYF) (Table 1). The cDNA inserts were directionally ligated into the vector pCMVsport6 (Invitrogen) digested with *Sal*I and *Not*I. The ligated vectors were transformed into ElectroMAX T1 DH10B cells (Invitrogen).

**Table 1:** EST libraries constructed for this study

Growth Conditions	Library Name	Insert size selection	Unique clones	Total unique ESTs	Insertless clones	Failed quality^1^	Passing to cluster	Small insert (100–700 bp)	Total number with BLAST hits^2^	Average diversity (passing clones)
Vegetative	CBYB	2–8 kb	4,992	9,888	2082	806	7000	789	299	58.8%
	CBYC	0.6–2 kb	1536	3071	164	523	2384	194	260	72.6%
										
Sporulation	CBYF	2–8 kb	1536	3072	1314	173	1585	305	123	82.3%
	CBYG	0.6–2 kb	1152	2304	834	186	1284	335	139	76.8%
	CCAW	None	4608	9216	340	1327	7549	3291	744	43.4%

^1 ^Comprised of ESTs of low quality sequence, low complexity, and presumptive contaminants.

^2 ^Evaluated with BLASTn against the non-redundant database

Library quality was assessed first by randomly selecting 24 clones and amplifying the cDNA inserts by PCR with the primers M13-F (5'-GTAAAACGACGGCCAGT) andM13-R (5'-AGGAAACAGCTATGACCAT). The number of clones without inserts was determined and 384 clones for each library were picked, inoculated into 384-well plates (Nunc; Nalge Nunc International, Rochester, NY) and grown for 18 hr at 37°C. After amplification by rolling-circle amplification (RCA), the 5' and 3' ends of each insert were sequenced using vector-specific primers (FW: 5'- ATTTAGGTGACACTA TAGAA and RV 5' – TAATACGACTCACTATAGGG) and Big Dye chemistry (Applied Biosystems; Foster City, CA). For each insert, the clone identification information was retained for the 3' and 5' sequence reads. An additional sporulation library was generated from the cultures producing conidia (library CCAW) although the inserts were not size selected, and they were directionally ligated into the *Sfi*IA/B sites of the vector pDNR-Lib (BD Biosciences). In total, bidirectional sequencing of each library generated 9888 ESTs from CBYB, 3072 from CBYC, 3072 from CBYF, 2304 from CBYG and 9216 from CCAW. All sequences were deposited into the GenBank dbEST database; accession numbers are provided for each EST in the Additional Materials.

### EST analyses and clustering

To trim vector sequences, common sequence patterns at the ends of ESTs were identified and removed. Clones were determined to lack inserts if ≥ 200 bases from the 5' end of the EST were identified as vector or if the insert was comprised of fewer than 100 bases of non-vector masked sequence. ESTs were then trimmed for quality with a sliding window trimmer (window = 11 bases). Once the average quality score in the window was below the quality threshold (Q15), the EST was split and the longest remaining sequence segment was retained as the trimmed EST. EST sequences with fewer than 100 bases of high-quality sequence were removed. ESTs were screened for the presence of polyA- or polyT-tails (which, if present, were deleted) and re-evaluated for length; ESTs with fewer than 100 bases were removed. ESTs consisting of more than 50% low-complexity sequence were removed from the final set of usable ESTs. If an EST required re-sequencing, the longest high-quality EST was retained. Sister ESTs or end-pair reads were categorized as follows: if one EST was insertless or a contaminant, then, by default, the sister EST was categorized as the same. However, each sister EST was treated separately for complexity and quality scores. Finally, EST sequences were compared against the GenBank nucleotide database by BLAST [22] to identify contaminants; undesirable ESTs such as those matching non-cellular sequences were removed.

For clustering, ESTs were evaluated with MALIGN [23], a kmer-based alignment tool that clusters ESTs based on sequence overlap (kmer = 16, seed length requirement = 32, alignment ID >= 98%). Clusters of ESTs were further merged based on sister reads using double linkage, which requires that two or more matching sister ESTs are in each cluster to be merged. EST clusters were then assembled using CAP3 to form consensus sequences. Clusters may contain more than one consensus sequence for various reasons (e.g., clone has long insert, clones are splice variants, consensus sequences are erroneously assembled). Cluster singlets are clusters of one EST, whereas CAP3 singlets are single ESTs that had joined a cluster but during cluster assembly were isolated into a separate consensus sequence. ESTs from each separate cDNA library were clustered and assembled separately, and subsequently the entire set of ESTs from all five cDNA libraries was clustered and assembled together with an external cDNA library (designated EXTA) obtained in an earlier study [16]. A file containing all clusters, cluster singlets, and CAP3 singlets is available in the Additional Materials.

Annotation of ESTs with GO terms was done with Blast2Go [24]. First, sequences were evaluated with BLASTx against the NCBI nr (non-redundant) database with an E-value threshold of 10^-5^. From a total of 7120 clusters, cluster singlets, and CAP3 singlets, 2526 sequences had no blast hits. Out of the remaining 4594 sequences, 2208 (48.1%) were categorized into different gene ontology (GO) classes at level three organization. For most clusters containing multiple consensus sequences or cluster singlets, a single Blast hit was selected for annotation. For a few clusters, some consensus sequences and/or cluster singlets corresponded to distinctly different genes, possibly due to overclustering, and thus were included in the final analysis. A file containing the annotation data for each cluster is provided in the Additional Materials.

### Real-time quantitative PCR (qPCR)

Expression profiles of 16 selected ESTs (Table 2) were determined by real-time PCR. Reactions were performed in an MXP-3000 real-time PCR system (Stratagene). Each reaction (20 μl) contained 10 μl of QuantiTect SYBR^®^-green PCR Master mix (Qiagen), forward and reverse primers (500 nM of each), cDNA template, and nuclease-free water. PCR cycling conditions were 10 min at 95°C (1 cycle), 15 s at 95°C followed by 1 min at 60°C (40 cycles), and a melting curve of 1 min at 95°C followed by 30 s at 55°C and a final ramp to 95°C with continuous data collection (1 cycle) to test for primer dimers and non-specific amplification. Expression of genes was measured in triplicate and expression levels were calculated by the comparative Ct method (Applied Biosystems). The 18S rRNA sequence for *C. zeae-maydis *(GenBank #EU399178), obtained from a small number of ribosomal contaminants in the EXTA library that were excluded from clustering analyses, was used as the endogenous reference for normalization. The sequence of β-tubulin (*TUB2*; GenBank #EU402967) was obtained by amplifying genomic DNA from strain SCOH1-5 with primers TubF (AACAACTGGCCAAGGGTCACTA) and TubR (GTCGAAGATTTGCTGGGTGAGCTC).

**Table 2:** Sequences examined by qPCR

Putative function or identity	Cluster or singlet ID	Primers for qPCR (5' to 3')	
		
		Forward	Reverse
Glycine-rich cell wall protein	1302	ACAAGTACACGTTCCTCCAGCTT	CAGCTCCAGCTCCAGCATCAT
Unknown	4016	CCAGGCAAATCAGACGGACTCT	TTTCAGCGCGCCAATGACACATTC
Unknown	2170	GGTCGGTGGCATTGTCGATTT	AAGGCCAAGAACGCCGTGAA
Unknown	1101	CAGGTCTCGCCGGTGTTAGA	ATGACTTTCTCGCGTTGGCATGAATG
Trihydrophobin precursor	2189	GGCCTAGAAGCCACTACGCTAA	CTGCTGCCTCATCCCACTTCT
Conserved hypothetical protein	13	ATGGACACTGGTGCCGGTTT	CCCTGAGCAGCATCCATGAAGT
Blue light-induced gene 3 (*bli-3*)	2277	GAGAGCACTATTCTCCTGCTCTCAAG	TTGATGAGGCCAATTCTCGGATCCT
Polyketide synthase (*CTB1*)	834	GTCGGTATCACCTGCAGATGGA	CCTGGCCACAAGACGAAAGTC
Opsin (*nop-1*)	839	CCAGTCAATGTAGCGGGCAAAGTAG	TCTGTCCTACTTCGCCATGGCTA
Cryptochrome/photolyase	CBYB4938	AGTTCTGGGATTGCTGGACCGAAA	TCTCGCCACCTTTATGAGGCGAA
Phytochrome	217	GGAGGTAGAAATCAGTATCGCAGACT	CCGGTGATGACGGGTTCTGT
Cutinase	2054	TTCAGCTGGGAGAGCAGGATCTT	CGCAGAAGGCATGATTGAAGTACTTG
Catalase	908	CTTCGGCGGCAAGTTCGACTA	CAGTCTTGCGAGTCCGTCATCAAG
Regulator of xylanase activity (*xlnR*)	740	GCCTTGCAGCAGATAGATTCCGAAA	CTCCTCACAGTCCTTCATCTCTGCTA
Regulator of cutinase activity (*cf1α*)	661	GCTGGCGAGACAGGTCTTGATC	GATGCCGCAGAGGCTCAAAAG
Transposable element (*Zephyr*)	17	GACGCTCTCCGTAAAGGCAAGAAC	GGGATATATAGGCGGTTCCGGTAGA
β-tubulin (*TUB2*)	n/a^1^	GGCTGGTGAGTGGTGCGAAA	GCTCAACAGCGATCTGCGCA
18S ribosomal	n/a	CAGGCCTTTGCTCGAATACATTAGCAT	GGATGCCCCCGACTATCCCTATTA

^1 ^n/a = not applicable

## Results and discussion

### Construction and sequencing of cDNA libraries

During the interaction between *C. zeae-maydis *and maize, two key aspects of the disease cycle are colonization of host tissue and the production of conidia for secondary inocula. Although we are especially interested in identifying genes underlying host/pathogen interactions, a major drawback of constructing cDNA libraries from inoculated leaves is that a high percentage of ESTs are likely to correspond to plant rather than fungal genes. To circumvent this problem, we created cDNA libraries from sporulating cultures in early and late stages of conidiation (sporulation libraries) as well as vegetative cultures grown under a variety of conditions that support or repress cercosporin biosynthesis (vegetative libraries).

**Figure 1:**
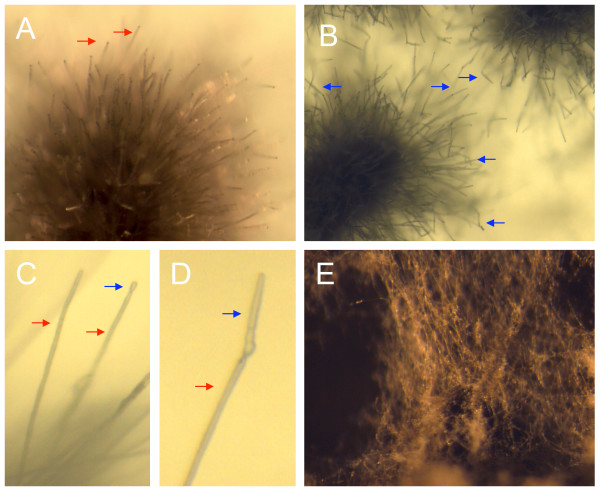
***Cercospora zeae-maydis *grown on V8 agar for sporulation libraries**. For library construction, cultures initiated from conidial suspensions were grown in constant darkness and RNA was extracted (A) three days and (B) five days after initiation, time points that correspond to early and late stages of conidiation, respectively. Conidiophores (red arrows) typically form two to three days after inoculation (C) and conidia (blue arrows) develop and mature over the next 48 hours (D). In contrast, cultures grown on the same medium in constant light produce few conidiophores or conidia (E).

In *C. zeae-maydis*, conidia are produced on long, slender conidiophores (Fig 1A) that give rise to solitary, hyaline conidia containing 5–7 septa (Fig 1B). We determined that, in constant darkness on V8 agar, cultures of *C. zeae-maydis *initiated from conidial suspensions are highly synchronous in conidiation, with conidiophores and nascent conidia visible by three days after inoculation (Fig 1C), and fully mature conidia visible five days after inoculation (Fig 1D). In contrast, cultures grown in constant light on V8 agar fail to produce conidiophores or conidia; instead, growth is exclusively vegetative (Fig 1E). For the sporulation libraries, we combined RNA from cultures harvested three and five days after inoculation with the goal of identifying genes involved in the early and late stages of conidiation. From two size-selected libraries (Table 2), we obtained 14,592 ESTs, of which 10,418 (71%) passed quality standards for clustering. Of the passing ESTs, 1585 (15%) came from the large-insert library CBYF, and 7549 (72%) originated from library CCAW, a non-size selected library having a relatively high proportion of small inserts (44%) and somewhat lower average sequence diversity (43.4%) than the other four libraries constructed for this study (Table 1).

**Figure 2:**
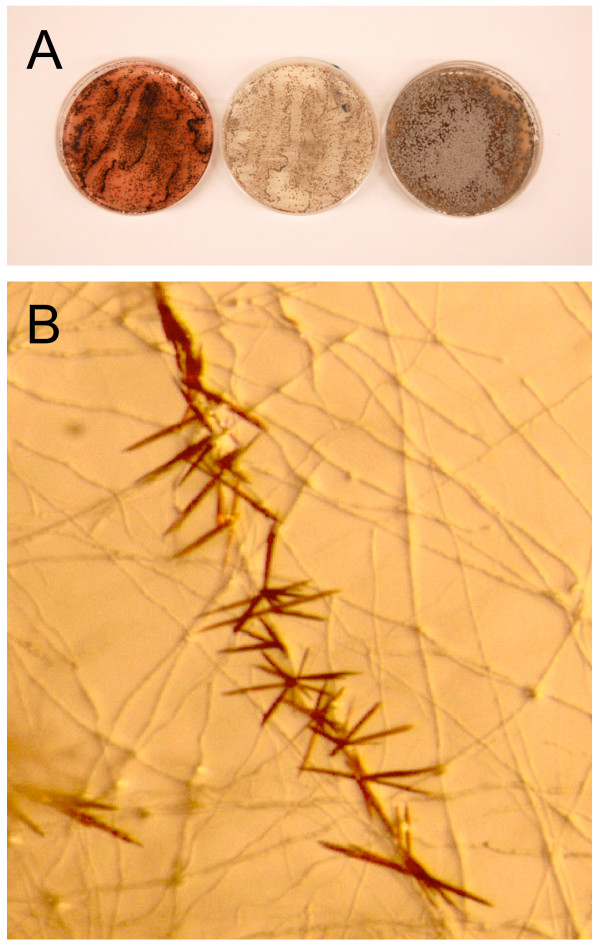
**Vegetative growth of *Cercospora zeae-maydis***. For construction of vegetative libraries (A), RNA was extracted from cultures grown in constant light on 0.2× PDA (left), on 0.2× PDA supplemented with 10mM ammonium phosphate (center), and on V8 agar (right). The red pigment visible in the culture grown on 0.2× PDA is cercosporin. When grown on 0.2× PDA (B), cercosporin frequently accumulates at high levels in the growth medium and forms crystals.

When grown on dilute (0.2×) PDA, *C. zeae-maydis *produces large amounts of cercosporin, which accumulates as a dark red pigment in the culture medium (Fig 2A) beginning after approximately three days of growth. Frequently, cercosporin is produced in large enough quantities to form crystals along hyphae of the fungus (Fig 2B). We used the accumulation of cercosporin as a visible marker to determine when to collect tissue for RNA extractions based on the hypothesis that genes involved in pathogenesis are induced concomitantly with the induction of cercosporin biosynthesis. We collected RNA from *C. zeae-maydis *growing on 0.2× PDA three and five days after inoculation with the intention of identifying genes expressed during the onset and after the accumulation of cercosporin biosynthesis. Additionally, to increase EST diversity, we collected RNA from cultures grown for five days on media that repress cercosporin biosynthesis irrespective of exposure to light, including V8-agar and 0.2× PDA supplemented with 10 mM ammonium phosphate, a preferred nitrogen source (Fig 2A). We constructed two distinct libraries from vegetative cultures (Table 2). From these two libraries, a total of 12,959 ESTs was obtained, 9384 (72%) of which passed quality standards and were clustered. The majority (75%) of the passing ESTs originated from the large-insert library CBYB.

ESTs from the five cDNA libraries described above were combined for clustering analysis. Among the 4088 clusters, we identified 7120 consensus identification sequences that primarily reflected non-overlapping sequencing reads due to a high percentage of large inserts in the CBYB library (data not shown). Fifteen clusters (0.4%) contained three consensus identification sequences, and six (0.1%) contained four or more, reflecting a combination of alternative transcript splicing as well as erroneous grouping of sequences (overclustering). To determine the distribution of cluster sequences between the two sets of conditions, we performed a cluster overlap analysis with the 4088 clusters containing two or more ESTs. A total of 1744 clusters were comprised of ESTs found exclusively in the vegetative libraries, whereas 1436 clusters were comprised of ESTs found exclusively in the sporulation libraries. Only 908 (18%) of the clusters were comprised of ESTs found in both the vegetative and sporulation libraries, thus indicating that the conditions selected for library construction have a substantial impact on the transcriptome of *C. zeae-maydis*.

### Sequence annotation and analysis

Fungal tissue from which cDNA libraries were constructed was obtained from cultures grown under a variety of conditions representing multiple stages of fungal development with the goal of obtaining a diverse collection of ESTs representing a range of molecular functions. ESTs were annotated according to Gene Ontology (GO) [25] guidelines with Blast2Go, a universal, web-based annotation application [24]. To ensure the highest recovery of GO terms, we submitted all 7120 consensus identification sequences derived from the 4088 clusters and 531 singletons for Blast2Go analysis. In total, 2526 sequences had no blast hits with an E value ≤ 10^-05^. Out of these, 2515 sequences contained one or more predicted open reading frames of at least 100 amino acids. The sequences that have coding potential but do not share significant homology to deposited sequences could represent conserved genes that are not yet described in other fungi or genes that are unique to *C. zeae-maydis*. Of the 4594 sequences with BLAST hits, 2208 sequences (48.1%) were assigned GO terms. To eliminate over-representation of GO terms, a single BLAST hit was included in the final analysis for each cluster unless multiple consensus sequences for a given cluster corresponded to remarkably different proteins. In total, 1471 clusters were assigned GO terms.

**Figure 3:**
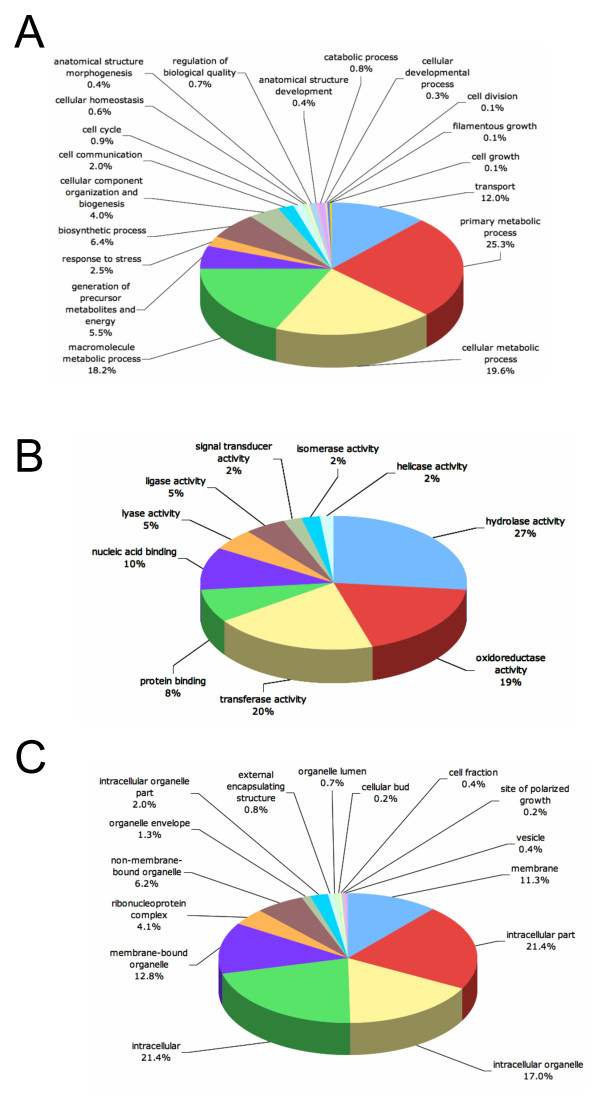
**Functional annotation of cluster consensus sequences based on Gene Ontology (GO) categorization**. Sequences were evaluated for their predicted involvement in biological processes (A), molecular functions (B), and cellular localization (C). Data are presented at level three GO categorization.

When analyzed by biological process, the majority of annotations (69%) were involved in metabolism, followed by transport (12%) (Fig. 3A). The remainder (19%) was distributed among several processes, including housekeeping functions, growth, and the regulation of development. Remarkably, despite our attempts to construct libraries enriched in genes regulating conidiation, only one sequence annotation was directly involved in asexual reproduction. We hypothesize that the apparent under-representation of conidiation-related genes reflects a general lack of knowledge of how fungi in general and *Cercospora *species in particular regulate asexual development, and that many conidiation-related genes in *C. zeae-maydis *reside among the 4912 sequences that either had no similarity to known sequences or could not be annotated with the GO system.

Hydrolases and oxidoreductases comprised over 45% of the total number of molecular functions identified by GO analysis (Fig 3B). Hydrolases, which utilize water molecules to break chemical bonds, perform a broad range of functions in fungi, including the extracellular digestion of complex carbon sources such as cellulose and other components of plant cell walls. Oxidoreductases catalyze the transfer of electrons between molecules and in fungi are involved in primary and secondary metabolism (including cercosporin biosynthesis) as well as the detoxification of compounds such as reactive oxygen species, superoxide and hydrogen peroxide. Intriguingly, these same compounds are frequently associated with the oxidative burst component of plant defense [26]. Although it is reasonable to propose that oxidoreductases of fungal foliar pathogens could be involved in detoxification of reactive oxygen species during pathogenesis, such a relationship has not been demonstrated. Sequences involved in signal transduction comprised 2% of the molecular functions identified (Fig 3B). Most of these genes were predicted to encode protein kinases, including 13 genes predicted to encode histidine kinases analogous to the two-component sensor histidine kinase family and three genes predicted to encode mitogen-activated protein kinases (MAPKs). The role of MAPKs in regulating morphology and virulence is well established in many fungi, including *C. zeae-maydis *[27,28]. In filamentous fungi and yeasts, histidine kinases trigger phosphorelay signaling mechanisms that interact with various MAPKs to regulate growth, differentiation, and virulence [29,30].

The vast majority of annotated sequences are predicted to encode intracellular proteins (Fig 3C). Considering the pathogenic lifestyle of *C. zeae-maydis*, we anticipated identifying a substantial number of secreted proteins, but found that limitations inherent to EST sequencing projects (e.g., 3' bias of sequence data, clones not corresponding to full-length transcripts) made predictions regarding secretion unreliable. However, nearly 1% of sequences were categorized by Blast2Go analysis as comprising external encapsulating structures, defined as any constituent of a structure that lies outside the plasma membrane and surrounds the entire cell [25]. Unlike bacteria, filamentous fungi generally produce highly hydrophobic proteins (collectively referred to as hydrophobins) rather than polysaccharide capsules as a protective barrier against the environment. The extent to which hydrophobins are involved in pathogenesis among filamentous fungi is not clear, but in *Magnaporthe grisea*, a hydrophobin encoded by *MPG1 *is required for the efficient induction of appressoria, possibly by mediating aspects of surface recognition [31].

Consistent with many fungal EST projects, a substantial number of sequences could not be annotated due to either a lack of BLAST hits or hits to uncharacterized fungal sequences [e.g., [32,33]]. Of the sequences with no BLAST hits, some fraction could be unique to *C. zeae-maydis*, whereas a significant percentage is likely to be too short to yield BLAST hits or correspond to untranslated regions of the mRNA (such as the 5' or 3' UTR). Of the sequences with BLAST hits, well over half could not be annotated due to a general lack of knowledge regarding the specific molecular functions of many fungal genes. For example, the genome of the closely related fungus *Mycosphaerella graminicola *is predicted to contain 11–12,000 genes, but to date, only ~30% have been annotated as to biological process, ~15% by cellular component, and ~40% by molecular function .

### Highly differentially expressed sequences

Consensus sequences consisting of ESTs found predominantly or exclusively in either vegetative or sporulation libraries could reveal molecular mechanisms involved in regulating fungal development, and the library-to-library distribution of ESTs corresponding to a single cluster offers at least a qualitative measure of gene expression. We designated consensus sequences comprised of at least 20 ESTs that were substantially enriched in either the vegetative or sporulation libraries (10-fold or greater distribution of ESTs in one set of libraries) to be highly differentially expressed sequences (HDESs). Twenty-six consensus sequences from the sporulation libraries met those criteria (Table 3). Of these 26 HDESs, fifteen (58%) were comprised of ESTs obtained exclusively during vegetative conditions, and seven were comprised of more than 100 ESTs. Many of the HDESs in the sporulation libraries were of unknown function (50%), with the next largest categories including genes predicted to be involved in protein synthesis (11%), mitochondrial sequences (11%), and components of primary metabolism (11%). The sequence most highly enriched during asexual development corresponded to prohibitin-1 of *Ajellomyces capsulatus*, the causal organism of histoplasmosis. Prohibitins are activators of Ras-induced signal transduction pathways that regulate growth and development in higher eukaryotes [34], but their molecular functions in fungi have not been established. In the vegetative libraries, only four sequences met the criteria of HDESs, all of which were comprised of 39 or fewer ESTs (Table 3). Three of the vegetative HDESs returned no hits after tBLASTx analysis against the nr database, thus indicating these sequences may be unique to *C. zeae-maydis*. The other vegetative HDES shared high levels of identity with fungal glutaminases, enzymes catalyzing the hydrolysis of glutamine to glutamic acid. Glutaminases play a role in the acquisition of nitrogen from less preferred sources, and a glutaminase from *A. nidulans *is subject to nitrogen metabolite repression [35]. The enhanced expression of a glutaminase on a nitrogen-poor medium such as 0.2× PDA is consistent with nitrogen metabolite repression.

**Table 3:** Consensus sequences highly enriched during either sporulation or vegetative growth

Cluster ID_consensus sequence	BLAST hit (gi #)^1^	E value	Annotation^2^	No. of ESTs in libraries	
				
				Sporulation	Vegetative
Enriched during sporulation					
35_8	154277410	6e-56	Prohibitin	387	3
4016_1	n/a	n/a	No hits found	325	1
35_7	73808014	6e-18	Exo-beta-1,3-glucanase	529	40
1101_1	n/a	n/a	No hits found	190	2
295_2	n/a	n/a	No hits found	211	6
2170_1	n/a	n/a	No hits found	133	0
11_11	39974277	4e-11	Conserved hypothetical protein	169	3
134_1	n/a	n/a	No hits found	67	0
2189_1	25091421	8e-13	Hydrophobin	63	0
2064_1	111064426	1e-20	Conserved hypothetical protein	85	2
13_1	156052246	3e-39	Conserved hypothetical protein	46	0
11_12	4572458	4e-34	Formate dehydrogenase	68	2
1356_1	46102748	9e-23	Conserved hypothetical protein	75	3
2201_1	n/a	n/a	No hits found	39	0
159_1	119173169	4e-85	Aquaglyceroporin	37	0
295_3	336895	6e-15	ATP synthase protein 9	50	2
2139_2	154281566	8e-56	Ribosomal protein L30	26	0
2156_1	145238173	9e-17	ATP synthase subunit Atp18	26	0
2324_1	46108711	1e-40	60S ribosomal protein L36	24	0
2255_1	154323144	3e-129	Enoyl-CoA hydratase	24	0
2134_1	5831429	3e-26	60S ribosomal protein L29	24	0
2277_1	5829297	6e-66	Blue light induced gene-3	23	0
2303_1	5828212	1e-49	Cytochrome c oxidase	22	0
294_1	145232507	2e-12	Conserved hypothetical protein	53	3
2154_1	n/a	n/a	No hits found	20	0
2301_1	84573599	9e-15	Conserved hypothetical protein	20	0
					
Enriched during vegetative growth					
540_1	71000414	0.0	Glutaminase	0	39
35_1	n/a	n/a	No hits found	0	26
1302_1	n/a	n/a	No hits found	0	23
35_3	n/a	n/a	No hits found	0	21

^1 ^gi = GenBank gene index number of most similar sequence obtained by tBLASTx.

^2 ^Projected annotation based on known function of all similar sequences obtained by tBLASTx with a threshold of 1e-5.

**Figure 4:**
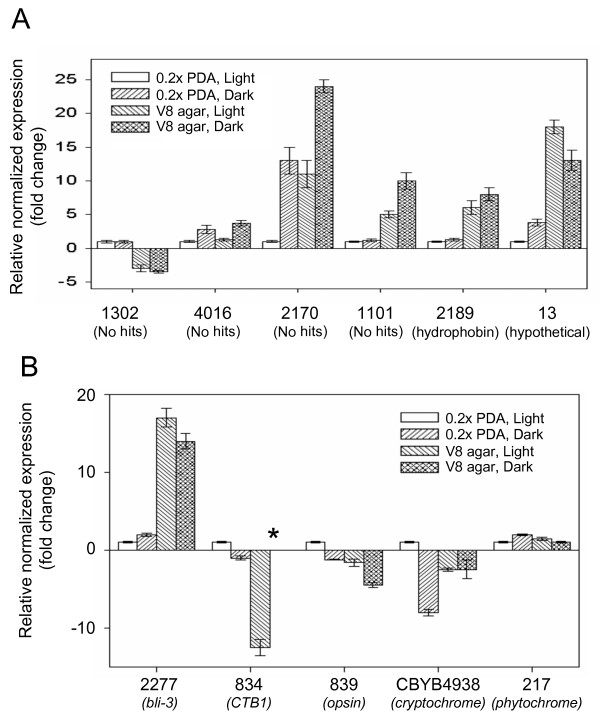
**Expression analysis of selected cluster consensus sequences**. (A) Expression of highly differentially expressed sequences from the vegetative and sporulation libraries was evaluated by quantitative PCR (qPCR) after three days of growth on 0.2× PDA in constant light, 0.2× PDA in constant darkness, V8 agar in constant light, and V8 agar in constant darkness. For each sequence, expression was normalized relative to expression during growth on 0.2× PDA in constant light. (B) Expression analysis of selected photoreceptors and putative light-regulated genes. (*) indicates not detected.

The enrichment of ESTs corresponding to a specific cluster sequence in one set of libraries over the other could reflect differences in fungal morphology and development or the effect of environmental conditions such as composition of growth medium. In this study, all cultures prepared for construction of vegetative libraries were grown in constant light on a variety of media, whereas all cultures prepared for sporulation libraries were grown in constant darkness on V8 agar. To correlate the occurrence and distribution of ESTs with levels and patterns of gene expression, we performed real-time quantitative PCR (qPCR) to evaluate seven HDESs in response to light and growth medium. Of five arbitrarily selected clusters that are highly enriched in sporulation libraries, three (1101, 2189, 13) showed substantially higher levels of expression on V8 agar compared to 0.2× PDA regardless of exposure to light, one (4016) appeared to be repressed by light, and another (2170) appeared to be regulated by both light and medium composition (Fig 4A). Of the four clusters enriched in vegetative libraries, at least one (1302) was regulated predominantly by medium composition (Fig. 4A). These analyses confirm that light and medium composition influence the transcriptome of *C. zeae-maydis *and that the distribution and frequency of EST occurrence in the two sets of libraries is a direct reflection of how genes are regulated in response to developmental and environmental cues.

### Photoreceptors and light-responsive genes

In many filamentous fungi, light affects diverse aspects of growth and development, including the formation of conidia, sexual reproduction, secondary metabolism, and circadian rhythms. Because light is a critical environmental regulator of pathogenesis in *C. zeae-maydis*, we focused on identifying genes involved in photoreception and light-dependent signal transduction. Two classes of photoreceptors have been identified in fungi: the heterodimeric blue-light-sensing *White collar *complex comprised of *White collar-1 *and *White collar-2 *[36] and the red-light-sensing phytochromes [37]. We identified a cluster highly similar to fungal phytochromes as well as sequences homologous to photoreceptors from higher eukaryotes that are uncharacterized in fungi, including blue/green light-sensing opsins, blue-light sensing phototropins, and blue-light sensing cryptochromes (Table 4). Additionally, we identified several sequences homologous to light-regulated genes, including circadian clock-regulated genes (Table 4). We were unable to identify a cluster corresponding to either *White collar-1 *or *White collar-2*, most probably due to their low basal levels of expression. Somewhat surprisingly, ESTs corresponding to *bli-3*, a blue-light induced gene from *N. crassa*, were found predominantly in the sporulation libraries; enrichment of light-induced genes would be unexpected in cultures grown in constant darkness.

**Table 4:** Putative photoreceptors and light-regulated genes found in EST libraries of *Cercospora zeae-maydis*

Cluster or singleton #	GenBank gi #^1^	BLAST e value	Annotation^2^	No. of ESTs in libraries	
				
				Sporulation	Vegetative
839	156057216	2e-99	Opsin	0	2
CBYB4938	121705009	3e-57	Cryptochrome/photolyase	0	1
3281	154291031	2e-80	Phototropin	0	2
217	39656354	0.0	Phytochrome	2	2
2277	5829297	6e-66	Bli-3	23	0
18	46137218	1e-11	Clock-controlled gene 6	14	35
404	32993638	0.0	Glyceraldehyde 3-phosphate dehydrogenase (clock-controlled gene 7)	8	8
					
2878	121713003	4e-16	Clock-controlled gene 8	2	0

^1 ^gi = GenBank gene index number of most homologous sequence obtained by tBLASTx.

^2 ^Based on comparative analysis of similar sequences obtained by tBLASTx with a threshold of 1e-5.

To investigate the transcriptional regulation of putative photoreceptors and light-regulated genes, we analyzed the expression of selected genes in response to light and growth medium. The sequence similar to members of the cryptochrome/photolyase family was more highly expressed in light than dark (Fig 4B) and, consistent with the distribution of corresponding ESTs (Table 4), the expression of the phytochrome-like gene did not appear to be affected by light (Fig 4B). Additionally, expression of the sequence similar to *bli-3 *appeared to be regulated primarily by growth medium (Fig 4B), thus explaining its enrichment in the sporulation rather than vegetative libraries.

### Sequences implicated in the regulation of development

Morphological differentiation and the regulation of development are complex processes in filamentous fungi and involve numerous genes and regulatory networks. We identified ten clusters corresponding to genes known to specifically regulate development and morphogenesis in other fungi (Table 5). Included among these clusters are several orthologs of genes regulating development in yeasts, including *zds1*, implicated in regulating multiple cellular events including sexual differentiation and morphology [38], *rcd1*, a key regulator of differentiation in response to nitrogen starvation [39], and *moc3*, a gene encoding a Zn-finger protein involved in sexual development and stress responses [40]. Additionally, we identified an ortholog of Ste12, a transcriptional regulator that in turn is regulated by MAP kinase signal transduction pathways [41]. Ste12 orthologs are well conserved among fungi and regulate various aspects of growth, differentiation, and pathogenesis [e.g., [42-44]].

**Table 5:** Sequences from EST libraries of *Cercospora zeae-maydis *implicated in fungal development

Cluster #	GenBank gi #^1^	BLAST value	Putative function (fungal homolog)^2^	No. of ESTs in libraries	
				
				Sporulation	Vegetative
545	67525274	5e-36	Conidiophore development protein (hymA)	0	4
1155	402369	1e-63	Regulator of conidiation (flbA)	0	4
1164	46125378	2e-55	Regulator of fruiting body formation (*nosA*)	0	4
1146	115387582	3e-109	Regulator of conidiation (nrc-2)	0	2
1217	154287709	1e-62	Regulator of development in response to nutrient availability (*rcd1*)	0	6
40	32995311	5e-85	Pleiotropic developmental regulator (fst12)	2	4

^1 ^gi = GenBank gene index number of most homologous sequence obtained by tBLASTx.

^2 ^Projected function based on comparative analysis of similar sequences obtained by tBLASTx with a threshold of 1e-5.

Because conidia play a key role in the propagation of diseases caused by *Cercospora *species, we are particularly interested in identifying genes involved in the regulation of asexual development. In *C. zeae-maydis *and many other filamentous fungi, conidia are borne on specialized structures termed conidiophores (Fig. 1A, C, D). However, the morphological characteristics of conidia and conidiophores vary widely among fungi, often to the extent that the size and shape of conidia and/or conidiophores form a basis for taxonomic identification of genera or species. Given the structural complexity of conidiophores and conidia as well as the extent to which conidiation is regulated by environmental cues, asexual development presumably requires the coordinated expression of many genes. However, relatively little is known at the molecular level regarding how fungi regulate conidiation. Much of the existing knowledge is derived from model fungi, such as *Aspergillus nidulans *and *Neurospora crassa*, which are only distantly related to *C. zeae-maydis*.

We identified several consensus sequences from the EST libraries corresponding to genes known to regulate conidiation in filamentous fungi (Table 5). In *A. nidulans*, the regulator of G-protein signaling *flbA *is required for asexual sporulation [45], and the Zn(II)2Cys6 transcription factor encoded by *nosA *that is required for the induction of sexual development is also transcriptionally upregulated during asexual development [46]. The exact molecular function of the protein encoded by *hymA*, also required for conidiophore formation in *A. nidulans*, is unknown [47]. In *Neurospora crassa*, an insertional mutant that constitutively initiated, but failed to complete, conidial development arose from disruption of *nrc-2*, a gene encoding a serine-threonine protein kinase [48]. Also, the putative green-light photoreceptor encoded by *nop-1 *regulates conidiation-specific gene expression in *N. crassa*, thus implicating the gene in fungal development [49]. Because of the complexity of conidiophore and conidial development and their relatively poor evolutionary conservation among taxonomic classes of fungi, further characterization of candidate genes involved in asexual development in *C. zeae-maydis *will require functional characterization such as targeted disruption.

### Pathogenesis-related sequences

As a foliar pathogen, *C. zeae-maydis *presumably produces a suite of enzymes during pathogenesis to facilitate the utilization of complex carbon sources, acquisition of nitrogen from non-preferred sources, and detoxification and/or avoidance of host resistance responses. We identified clusters encoding a wide variety of catabolic enzymes likely to be involved in leaf colonization, including cellulases, cutinases, cellobiases, xylanases, glucosidases, cellobiohydrolases, lipases, and proteases (Table 6). Additionally, we identified conserved transcription factors implicated in pathogenesis (Table 6), including *areA*, a central regulator of nitrogen metabolite repression and a key regulator of secondary metabolism in filamentous fungi [50], and homologs of *xlnR *and *ctf1a*, which encode conserved regulators of xylanase and cutinase expression, respectively [51,52]. Consistent with a dichotomy between infectious and reproductive growth, ESTs encoding pathogenesis-related sequences were found predominantly in the vegetative libraries.

**Table 6:** Pathogenesis-related sequences identified in EST libraries of *Cercospora zeae-maydis*

Cluster no.	GenBank gi #^1^	BLAST e-value	Putative function^2^	No. of ESTs in libraries	
				
				Sporulation	Vegetative
834	50080728	7e-124	Polyketide synthase involved in Cercosporin biosynthesis (*CTB1*)	0	3
1641	156446810	2e-80	Polyketide synthase	0	2
3502	40787371	1e-125	Polyketide synthase	0	1
859	121704362	3e-103	Hybrid non-ribosomal peptide synthetase/polyketide synthase	0	4
3456	119469758	6e-65	Non-ribosomal peptide synthetase	0	1
1346	121714834	1e-161	Extracellular lipase	2	4
1982	119472133	3e-91	Cellulase	0	1
2054	121716183	1e-51	Cutinase	0	2
908	164425479	9e-126	Catalase	0	19
740	154317444	6e-97	Regulator of xylanase activity (*xlnR*)	0	8
661	67541772	7e-135	Regulator of cutinase activity (*cf1α*)	0	7
3591	12082799	4e-43	Nitrogen response factor (*areA*)	0	1
1982	119472133	3e-91	cellobiohydrolase	0	3
1254	115389439	4e-80	β-glucosidase	0	7
527	13816425	7e-44	β-glucosidase	0	7
641	115389439	7e-135	β-glucosidase	0	5
136	74054463	2e-107	β-glucosidase	3	12
1663	15982667	3e-98	β-glucosidase	1	3
1059	156061937	4e-103	α-glucosidase	0	6
1855	1552175	8e-69	xylanase	0	4

^1 ^gi = GenBank gene index number of most homologous sequence obtained by tBLASTx.

^2 ^Projected function based on comparative analysis of similar sequences obtained by tBLASTx with a threshold of 1e-5.

**Figure 5:**
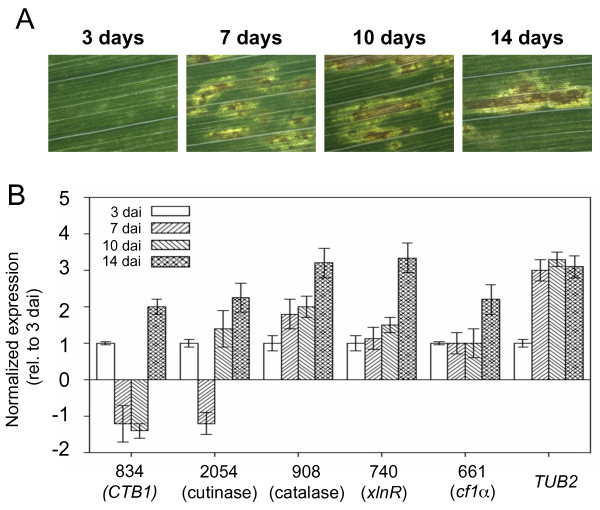
**Expression analysis of putative pathogenesis-related sequences during infection**. (A) Infected maize leaves were collected three, seven, ten, and fourteen days after inoculation. (B) Real-time quantitative PCR was performed to analyze expression of selected fungal genes during pathogenesis.

Infection of leaves by *C. zeae-maydis *progresses through distinct stages, including spore germination, appressorium formation, penetration of leaves, and a transition to necrotrophic growth. Underlying each of these stages are unique molecular interactions reflected at least in part through modulation of gene expression. To establish the expression profiles of pathogenesis-related genes during distinct stages of leaf colonization, we performed qPCR on leaves three, seven, ten, and 14 days after inoculation (Fig 5A). The expression of two transcriptional regulators of extracellular enzymes involved in catabolism and a putative cutinase gradually increased during colonization, as did the expression of a putative catalase (Fig 5B), which suggests that these genes are involved in colonization of host tissue.

Many fungal secondary metabolites, including cercosporin, are polyketide compounds formed by the head-to-tail condensation of acetate molecules as catalyzed by polyketide synthases. Recently, a gene cluster encoding a group of biosynthetic genes required for cercosporin biosynthesis was identified in *Cercospora nicotianae*, a foliar pathogen of tobacco [15]. The cluster contains a polyketide synthase (*CTB1*), disruption of which abolishes cercosporin biosynthesis [53], as well as other coordinately regulated genes such as oxidoreductases hypothesized to catalyze specific steps in the biosynthesis of cercosporin [54]. Consistent with established patterns of cercosporin biosynthesis in culture, the *C. zeae-maydis *homolog of *CTB1 *was induced by light on 0.2× PDA and was repressed by V8 agar irrespective of exposure to light (Fig 4B).

Although cercosporin is known to function as a virulence/pathogenicity factor in many *Cercospora *species, the dynamics of cercosporin biosynthesis during pathogenesis are largely unknown. To explore this question, we monitored *CTB1 *expression during colonization of leaf tissue. Although expression of *CTB1 *increased two-fold by 14 days after inoculation, it was somewhat surprising that expression of *CTB1 *changed little from 3–10 days after inoculation (Fig 5B). During this time, the fungus makes its initial penetration of mesophyll tissue and, as reflected by the visible development of lesions, commences necrotrophic growth. The absence of *CTB1 *induction during these stages of pathogenesis suggests that other virulence/pathogenicity factors may play a greater role in the initial colonization of leaf tissue.

Somewhat surprisingly, no other sequence similar to genes in the cercosporin biosynthesis (*CTB*) cluster was found among the ESTs obtained in this study. Among fungi that produce a given secondary metabolite, the underlying gene clusters are generally highly conserved, making it unlikely that *C. zeae-maydis *possesses a fundamentally different mechanism responsible for cercosporin biosynthesis. Rather, the most likely explanation for the absence of other *CTB *homologs from the EST dataset is that the cultures from which the vegetative libraries were produced represented a variety of growth conditions, not all of which supported cercosporin biosynthesis; therefore, the relative concentration of mRNAs corresponding to *CTB *genes was diluted. We hypothesize that more extensive sequencing of the vegetative libraries would lead to the identification of homologs of *CTB *genes such as those identified in *C. nicotianae*.

### Identification and characterization of Zephyr, a novel transposable element

Among the ESTs highly represented in the vegetative library compared to the sporulation library, we identified a sequence highly similar to members of the Ty3/Gypsy family of long terminal repeat (LTR) transposons, including *Grasshopper *from *Magnaporthe grisea *[55], *REAL *from *Alternaria alternata *[56], and *Skippy *from *Fusarium oxysporum *[57]. Members of the family typically contain two long, partially overlapping open reading frames encoding a protein similar to retroviral structural proteins and a poly protein containing protease, reverse transcriptase, RNaseH, and integrase domains [58]. The retroelement identified in this study, designated *Zephyr*, is comprised of four clusters of 1749, 3664, 1028, and 1707 bp as well as four cluster singlets consisting of 110, 766, 745, and 252 bp. A conceptual translation of the 3664-bp cluster results in a protein of 1221 amino acids that corresponds to the poly protein of the element and is highly similar to Ty3/gypsy elements found in *Magnaporthe grisea *and other filamentous fungi, including the closely related fungus *Mycosphaerella graminicola *(data not shown). To date, only one other retroelement has been identified in *C. zeae-maydis*: *Malazy*, a degenerate, presumably non-coding member of the gypsy family [59] that shares substantial identity with *Zephyr *at the nucleotide level. Because the numerous premature stop codons found in *Malazy *are absent from *Zephyr *and EST evidence indicates *Zephyr *is an active element, we hypothesize that *Malazy *represents a defective/inactivated descendent of *Zephyr*.

Transposition of retroelements in fungi can be induced by a variety of biotic and abiotic stresses as well as morphological changes such as sexual reproduction. However, activation of transposable elements is relatively rare during the normal growth and development of most organisms, including filamentous fungi [60]. Therefore, it is somewhat surprising that ESTs representing the polyprotein-encoding region of *Zephyr *are highly enriched in the vegetative libraries (34 ESTs in vegetative libraries compared to two in sporulation libraries). To further characterize the regulation of *Zephyr*, we profiled its expression during growth of *C. zeae-maydis *under a variety of environmental conditions. When evaluated by qPCR, cycle threshold values were low (< 20), thus indicating high levels of expression. Consistent with the distribution of ESTs in vegetative and sporulation libraries, the pol polyprotein-encoding region of *Zephyr *was expressed nearly 4-fold greater during growth on 0.2× PDA in light than on V8 agar in darkness. These results suggest that *Zephyr *is an actively transcribed element that is regulated by growth medium and possibly by light. Although EST and qPCR data indicate that *Zephyr *is highly expressed, further studies will be required to verify the transposition of the element.

Currently, little is known regarding the molecular mechanisms controlling the activity of transposable elements in fungi. Transposable elements have been implicated as a driving force behind genetic diversity; their activation in response to environmental stress is hypothesized to be a mechanism of adaptation, and consequently, genomic evolution [61]. Because *Cercospora *is believed to be a largely asexual genus, transposition of elements such as *Zephyr *could be a driving force behind the remarkably high level of host-specific speciation that has evolved among *Cercospora *species.

## Conclusion

By generating ESTs from vegetative and sporulating cultures of *C. zeae-maydis*, we identified novel genes involved in a wide range of biological processes. Functional annotation and expression profiling implicated subsets of genes in pathogenesis and conidiation. Consistent with the crucial role light plays in host-pathogen interactions between *C. zeae-maydis *and maize, we identified a large number of photoreceptors and light-regulated genes, plus *Zephyr*, a novel, highly expressed transposable element. We conclude that light plays a key role in the dichotomy between vegetative and reproductive growth in *C. zeae-maydis *and that future characterization of the underlying molecular mechanisms will contribute significantly to the fundamental understanding of how fungi respond to light.

## Authors' contributions

BHB participated in the design and execution of this project. He performed the microscopy, performed RNA extractions, assisted with the functional annotations, performed the quantitative PCR, and drafted the manuscript. BD performed the functional annotations and assisted with drafting the manuscript. EAL coordinated the construction and sequencing of cDNA libraries and performed the cluster overlap analysis. GHJK and SBG conceived of this project, participated in its design, and wrote the proposal that allowed it to occur. LDD also conceived of this project, played a major role in its design and coordination, and helped prepare the manuscript. All authors read and approved the final manuscript.
